# Supplementary material for: Anti-Oxidized Self-Assembly of Multilayered F-Mene/MXene/TPU Composite with Improved Environmental Stability and Pressure Sensing Performances
Source: Polymers (Basel). 2024 May 9;16(10):1337. doi: 10.3390/polym16101337 (PMC11125229; doi:10.3390/polym16101337)
Supplement: Supplementary file 1 [file polymers-16-01337-s001.zip › polymers-2991517-supplementary.pdf]

Supporting Information

Anti-Oxidized Self-Assembly of Multilayered F-Mene/MXene/TPU Composite with Improved Environmental Stability and Pressure Sensing Performances

Zhong Zheng, Qian Yang, Shuyi Song, Yifan Pan, Huan Xue and Jing Li *

Hubei Key Laboratory of Modern Manufacturing Quantity Engineering, School of Mechanical Engineering, Hubei University of Technology, Wuhan 430068, China; zhengzh@hbut.edu.cn (Z.Z.); rain20220204@163.com (Q.Y.); songshuyi1016@163.com (S.S.); yuunagi0824@outlook.com (Y.P.); stonemechanics@163.com (H.X.)

* Correspondence: lijing@hbut.edu.cn; Tel.: +86-027-59750012

Figure S1 shows the SEM images of TPU fiber mats prepared by electrospinning. The fiber surface is smooth and continuous, with a diameter of about ~1 micron. The fibers are interwoven to form an interconnected and porous structure, and the pore size is at the micron level. The prepared TPU fiber mat has a large specific surface area and high porosity. The large specific surface area means that the anchoring area of MXene increases, and the high porosity is conducive to the continuous resistance response of the sensor in the large pressure load range.

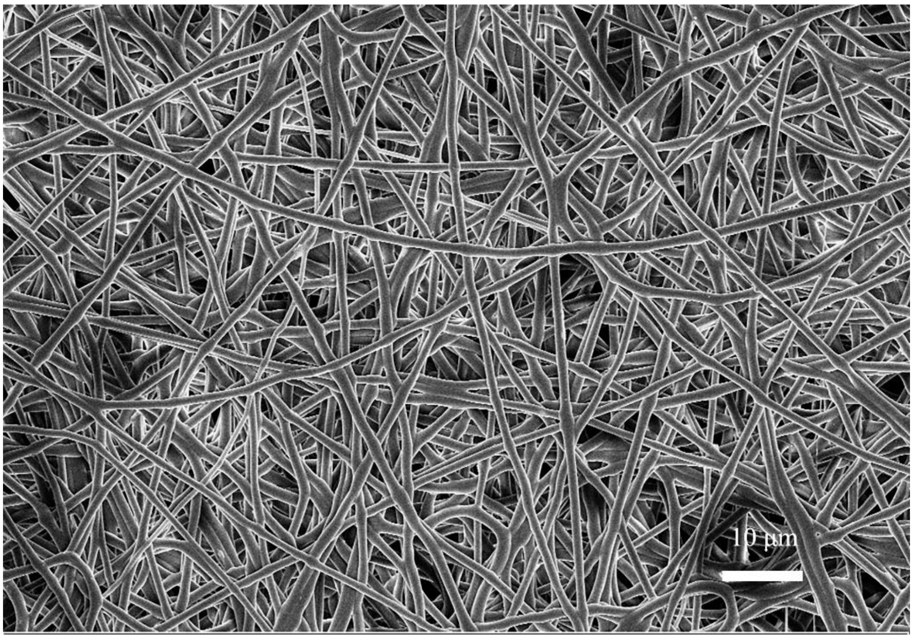

Figure S1. SEM image of the original TPU fiber mat.

Figure S2 is the local magnification of the f-MXene/MXene/TPU fiber mat. It can be observed from the figure that the smooth TPU fiber becomes rough, indicating that the f-MXene, MXene conductive material is attached to the TPU fiber while retaining the porous network structure of the TPU fiber.

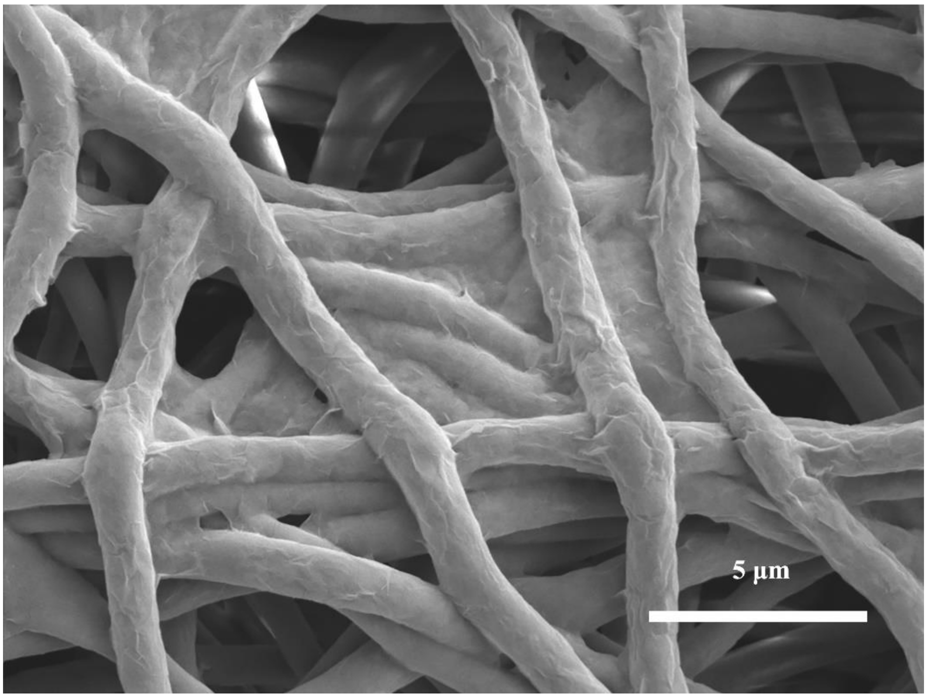

Figure S2. SEM image of f-MXene/MXene/TPU fiber mat.
